# Acute Graft-*Versus*-Host Disease, Infections, Vascular Events and Drug Toxicities Affecting the Central Nervous System

**DOI:** 10.3389/fimmu.2021.748019

**Published:** 2021-10-06

**Authors:** Janaki Manoja Vinnakota, Robert Zeiser

**Affiliations:** ^1^ Department of Medicine I - Medical Centre, Faculty of Medicine, University of Freiburg, Freiburg, Germany; ^2^ Faculty of Biology, Albert-Ludwigs-University, Freiburg, Germany; ^3^ Signalling Research Centres BIOSS and CIBSS – Centre for Integrative Biological Signalling Studies, University of Freiburg, Freiburg, Germany

**Keywords:** GvHD, central nervous system, inflammation, drug toxicity, microglia, T cells

## Abstract

Allogeneic hematopoietic cell transplantation (allo-HCT) is a curative therapy for patients with hematological malignancies. Acute Graft *versus* host diseases (GVHD) is a major immune mediated side effect of allo-HCT that can affect the central nervous system (CNS) in addition to post-allo-HCT vascular events, drug toxicity or infections. Here we summarize and discuss recent preclinical data on the CNS as a target of acute GVHD and the known mechanisms contributing to neurotoxicity with a focus on microglia and T cells. We also discuss open questions in the field and place the findings made in mouse models in a clinical context. While in mice the neurological deficits can be assessed in a controlled fashion, in patients the etiology of the CNS damage is difficult to attribute to acute GVHD *versus* infections, vascular events, and drug-induced toxicity. Ultimately, we discuss novel therapies for GVHD of the CNS. Our understanding of the biological mechanisms that lead to neurotoxicity after allo-HCT increased over the last decade. This review provides insights into CNS manifestations of GVHD *versus* other etiologies of CNS damage in mice and patients.

## Introduction

Acute graft-*versus*-host disease (GVHD) is a life-threatening complication after allogeneic hematopoietic cell transplantation (allo-HCT). About 50% of the patients with severe acute GVHD fail to respond to corticosteroids, and steroid-refractory severe GVHD has a dismal prognosis with a 1-year survival rate of less than 20% (1). GVHD was classically considered to involve the skin, intestinal tract and liver, which was termed as “tissue tropism of acute GVHD”. The target organs of acute GVHD are affected by commensal bacteria that populate these locations and that may migrate through damaged epithelial barriers (2) and activate intestinal epithelium (3), neutrophils (4, 5), dendritic cells, macrophages and monocytes (6). The observation that non-sterile triggers of tissue damage such as ATP (7, 8) or uric acid (9) may contribute to GVHD support the concept that also other organs with less commensal bacteria can be affected by GVHD. There is increasing evidence that the effects of acute GVHD are not limited to the three classical target organs, but can also occur in the central nervous system (CNS). Neurological complications were reported in 10% of the patients undergoing autologous (auto)-HCT while over 80% of allo-HCT patients experienced neurological complications at some time point (10–12) which indicates that not only the toxicity but also the allo-reactive effect of the donor immune system may contribute to neurological complications. Clinical manifestations of CNS-GVHD include seizures, reduced vision and cognitive impairment. The symptoms can resemble for example multiple sclerosis or Guillain-Barre syndrome. Risk factors for neurological complications during acute GVHD are diverse. Female gender, high doses of total body irradiation (TBI), myeloablative high dose chemotherapy-based conditioning, infections and preexisting cerebrovascular disorders are major risk factors for the development of neurological complications after allo-HCT (13–15). CNS-GVHD though considered a rare entity, significantly affects the mortality and quality of life in allo-HCT patients (13). In this review, we provide an overview on the cell types affected by CNS-GVHD and we discuss the diverse clinical manifestations of the disease as well as infections, vascular events and drug toxicities affecting the CNS.

## Studies on CNS-GVHD in Preclinical Models

Preclinical studies using mouse models of acute GVHD showed that the transfer of allogeneic T cells caused CNS infiltration by effector memory T cells (16). The allogeneic T cells infiltrated different regions of the CNS including the meninges, vasculature and parenchyma while a comparable T cell infiltration was not observed when only syngeneic T cells were transferred (16). Evidence for CNS-GVHD was not restricted to the murine model, as other investigators reported that CNS infiltration by CD8^+^ T cells was a key feature of GVHD in non-human primates (17). Conversely, treatment of primates with immune-prophylaxis after allo-HCT reduced the abundance of T cell infiltration into the brain (17). These findings indicate that the donor T cells manage to infiltrate the CNS despite its anatomical location and immune privilege. Therefore, immune responses may evolve differently from peripheral tissues. This infiltration by T cells is likely due to disruption of the blood-brain-barrier, which under normal conditions controls the influx of immune cells into the CNS.

**Figure 1:**
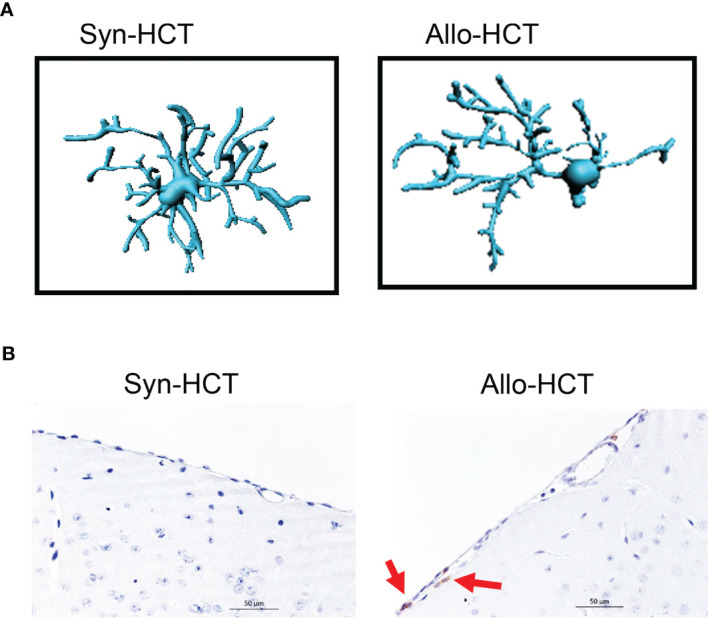
**(A)** Microglia morphology in the CNS of mice undergoing syn-HCT or allo-HCT as previously reported (26). **(B)** Infiltration of T cells (brown) in the CNS of mice undergoing syn-HCT or allo-HCT.

Though T cells play a central role for the induction of acute GVHD, other cell types also contribute to the disease. Studies reported an increase in the expression of MHC class I and II molecules in the CNS in a rat model of GVHD. Immunohistological studies revealed increased expression of host MHC in parenchymal and vascular regions along with increased infiltration of T cells (18). In line with the findings, a fivefold increase in the MHC-II expression was observed in a CD45^lo^CD11b^+^ microglial population which further re-iterates the involvement of microglia in CNS-GVHD pathogenesis (19). Microglial activation was not only observed in inflammatory disease of the CNS but also in several neurodegenerative diseases including Parkinsons disease and Alzheimers disease (20). Host derived IL-6 and Indoleamine 2,3 Dioxygenase-1 (IDO-1) were shown to regulate the behavior patterns and inflammation in the CNS during acute GVHD (21). Microglia and macrophages were activated and increased the production of IDO-1 which thereby resulted in behavioral deficits in a murine model of GVHD (22). Interestingly, IL-6R inhibitor treated mice had decreased infiltration of CD4 and CD8 T cells and reduced production of pro-inflammatory cytokines in CNS. Recent clinical studies showed that downstream signaling of IL-6R *via* JAK2/STAT reduced acute and chronic GVHD in patients (23–25). We have previously shown that microglia plays a central role in acute GVHD-induced neurotoxicity (26). Acute GVHD caused an amoeboid phenotype of microglia with reduced branching points and dendrites when compared to the syngeneic HCT controls in a murine model of GVHD (**Figures 1A, B** **)**. Microglia cells that were activated during acute GVHD exhibited increased TNF expression and activated the downstream TAK/MAPK signaling. Therapeutic inhibition of TAK1 signaling by takinib reversed the microglial activation and T cell infiltration (26). Additionally the GVHD induced neurocognitive defects reduced in mice treated with takinib, suggesting a novel potential therapeutic avenue for acute GVHD of the CNS.

Consistent with the neurocognitive defects observed in mice developing GVHD, neuronal damage in the CNS was reported (16). Allogeneic T cells infiltrating the CNS induced apoptosis of neurons and neuroglia, which limited the cognitive and exploratory function in a murine model of GVHD (16). In line with the findings, an increase in the expression of c-fos was noted in several cortical regions including occipital and olfactory regions in a rat GVHD model (27). In contrast, such inflammatory effects were not observed upon transfer of syngeneic T cells (27).

**Figure 2:**
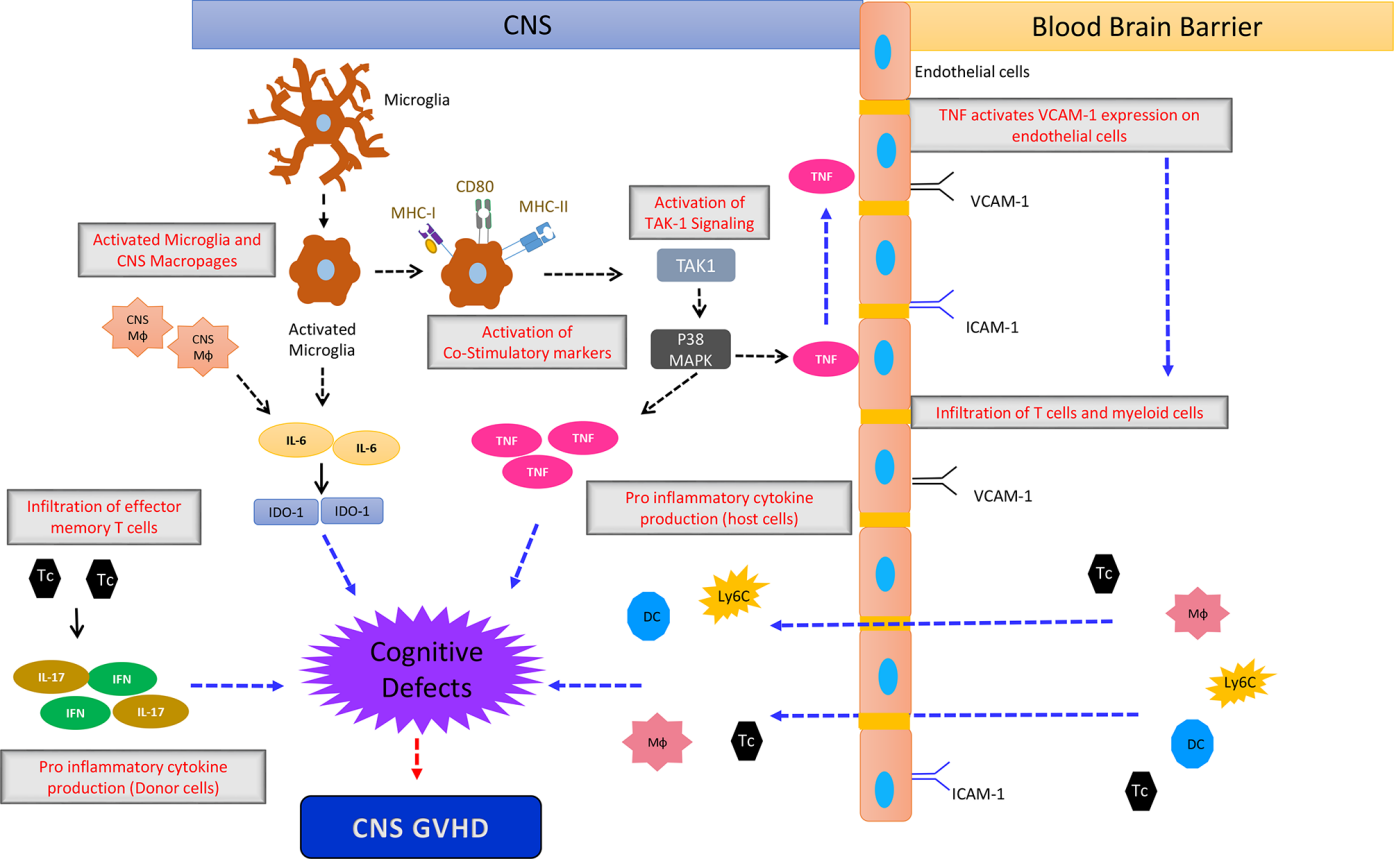
The simplified sketch shows the proposed mechanism how CNS-GVHD evolves and contributes to neuronal damage ultimately leading to cognitive deficits. An initial event is the activation of microglia by stimuli that are not well characterized so far, being most likely damage associated molecular patterns (DAMPs) and pathogen associated molecular patterns (PAMPs). Activated microglia upregulates MHC I and II as well as CD80 leading to increased T cell priming. Additionally microglia- and macrophage-derived IL-6 impacting IDO-1 induces neurological defects, leading to the clinical picture of CNS GVHD. TNF derived from microglia has direct neurological toxicity. Donor T cells polarized towards Th1 and Th17 contribute to CNS GVHD as well as macrophages, monocytes and DC from the periphery. Mф, Macrophage; DC, Dendritic Cells; Tc, T cells; Ly6c^+^ cells, Monocytes.

Multiple effects involving endothelial damage, T cell transmigration, cytokine production and ultimately neuronal damage are involved in CNS-GVHD (**Figure 2**).

## Human Studies on CNS-GVHD

Consistent with findings in preclinical models, human brain analysis of female sex-mismatched bone marrow transplant recipients have identified donor (Y-chromosome^+^) derived cell infiltrates (28). In addition to this, lymphocytosis was noticed in CSF together with encephalitis with increased infiltration of T cells and gliosis with no signs of infection further confirming the occurrence of CNS-GVHD (29, 30).

Neurological deficits and MRI findings have been reported in patients developing GVHD (31).

The clinical picture of acute GVHD is often connected to neurological deficits in patients, morphological CNS white matter changes detectable by magnetic resonance imaging and intraparenchymal lymphocytic infiltration of the brain upon autopsy (31, 32). In line with the findings, studies also reported neurological deficits including drowsiness, dysphoria, right dazedness and MRI findings of abnormal cerebra gyrus swelling, corpus signal, diffused white matter regions (33). Biopsy studies on GVHD brains showed axonal depletion representative of demyelination disease in a patient. CNS-GVHD is quite heterogeneous and case dependent with patients most frequently reported with delusion, hemiparesis, temporary unconsciousness and psychomotor agitation with neither T cell infiltration to the CNS nor relapse of malignancy (14, 34). On contrary, some patients also developed metabolic encephalopathy with neurological deficits ranging from vision loss, confusion to coma and death (15).

Autopsy studies revealed an increase of Iba-1^+^ myeloid cells in the CNS of patients with GVHD when compared to the allo-HCT patients without GVHD. In addition to this the microglia from CNS-GVHD patients had increased expression of TNF (26).

Due to the rarity of CNS-GVHD and the difficulty to distinguish the disease from other mediators of CNS toxicity, biomarkers to identify CNS-GVHD would be highly desirable. IgG index in the CSF is an indicator of neurological disorders like multiple sclerosis, intrathecal inflammation (35, 36). Another study indicated that Blood Brain Barrier (BBB) impermeability, IgG –Synthesis index are early indicators of CNS demyelination (37). In addition to this, increased BBB permeability, elevated myelin basic protein in blood and CSF are some of the immune markers that could be tested for their validity as biomarkers for CNS-GVHD (36). Identifying the immune biomarkers that predict damage to neurons, glial cells and myelin membranes may help diagnose CNS-GVHD. Patients with CNS-GVHD were reported to respond to high dose corticosteroids, intravenous immunoglobulin treatments, immunosuppressive medications including methotrexate and etoposide (38). Chronic CNS-GVHD is a late complication of allo-HCT and clinical manifestations may include myasthenia gravis, myositis, demyelination, angiitis (39, 40). Patients can also present with stroke-like episodes, lacunar syndromes, multiple sclerosis-like presentations or encephalitis (30). The diagnosis of chronic CNS-GVHD is often challenging (41). The NIH Consensus Conference on criteria for clinical trials in chronic GVHD delineated three types of chronic CNS-GvHD: cerebrovascular disease, CNS demyelinating disease, and immune-mediated encephalitis (41). The NIH consensus on criteria for clinical trials in chronic GVHD recommended that the diagnosis of chronic CNS-GVHD should be made only when other organs are affected by GVHD and other neurological differential diagnoses are excluded (41). Differential diagnoses of chronic CNS-GVHD include in particular drug-induced toxicities or opportunistic infections.

## Non-GVHD Related Causes for Neurological Symptoms After Allo-HCT

Neurological complications after allo-HCT can have multiple etiologies such as infections, vascular events and drug-induced toxicities.

After allo-HCT, patients are immunodeficient and therefore highly susceptible to a variety of opportunistic infections caused by either bacteria, fungi or viruses, which can also affect the CNS. Acute GVHD further increases the risk of opportunistic infections, which lead to neurological complications in some patients (42). CSF analysis of patients undergoing allo-HCT revealed the presence of cytomegalovirus (CMV), Epstein Bar (EBV), Human Herpes virus-6 (HHV-6), HHV-8, toxoplasma infections among others (43). Diffuse microglial hyperplasia and microglial nodular encephalopathy were reported in some patients, which indicates microglial activation in response to infectious complications during GVHD (15). Meningoencephalitis induced by Aspergillus species was observed in children and adults undergoing allo-HCT with an overall incidence rate of up to 30% (15, 44). Cerebral aspergillus infections can cause stroke like manifestations with focal deficits (45). Infections related to candida species were reported in allo-HCT patients with neurological complications ranging from vasculitis to hemorrhagic abscess (46). Bacterial infections also account for major neurological complications after allo-HCT, e.g. CNS infections with streptococcus and staphylococcus (15). Klebsiella, E coli and Listeria monocytogenes were reported to cause meningitis and brain stem encephalitis in allo-HCT patients. Toxoplasma gondii encephalitis is a rare infection after allo-HCT, mostly reported in countries with high prevalence rates of the toxoplasma (47, 48). Neurotoxoplasmosis is characterized by the presence of grey and white matter abscesses and can be diagnosed by CT or MRI scans (49). Patients undergoing allo-HCT are exposed to a variety of viruses that lead to viral encephalitis further governing the mortality and morbidity rates. HHV-6, EBV, Herpes simplex virus, CMV, John Cunningham (JC) virus, varicella zoster virus, and adenovirus are the commonly reported viral infections leading to neurological complications in GVHD patients. Progressive multifocal leukoencephalopathy is a progressive demyelinating disorder caused by JC virus primarily affecting oligodendrocytes in response to monoclonal antibodies (50). Restoration of anti-viral immune responses is the only available option for treating JC virus related infections, although tapering the immunosuppression was unsuccessful in reversing the neurological deficits in a fraction of patients (51). In addition to this, a positive correlation between CD8^+^ T cells in the CNS and JC virus infected glial cells was reported (52). HHV-6 induced encephalitis is a serious complication observed mostly within 24 days of allo-HCT (53, 54). Patients with high levels of HHV-6 DNA in their plasma are at an increased risk of developing neurological deficits that include epilepsy, delirium, and cognitive impairment (53, 55). Pediatric patients receiving haploidentical CD45RA T cell depleted grafts presented with a high rate of HHV-6 induced encephalitis (56). Similarly, HSV related infections can affect the CNS (57). Unlike HSV, VZV infection typically affects multiple region in the CNS and the common manifestations include myelitis and vascular encephalitis. Post-transplant lymphoproliferative diseases driven by oncogenic EBV pose considerably high risks post allo-HCT (58). The infections caused by EBV are early onset and mostly donor derived and risk factors include intensity of immunosuppression and high-grade GVHD (59). The manifestations are very similar to CNS lymphomas with hypercellularity, necrosis and hemorrhages (60). CMV infections in either lungs or CNS are often associated with extremely high mortality rates in allo-HCT patients. CMV infection of the CNS is typically a late onset disease and is associated with encephalitis or polyradiculopathy (61). Umbilical cord transplantation and prolonged T cell depletion are the major risk factors associated with CMV encephalitis (62). Histological manifestations of the CMV include viral inclusion bodies in the CNS commonly referred as owls eye inclusions (63). In some patients the viral load of CMV in the CSF was higher than in the peripheral blood indicating the significance of monitoring the CMV copy levels in the CSF when CNS involved by CMV reactivation is suspected (63, 64). Allogeneic virus-specific T cells were shown to be effective against CMV and EBV (65–67) and could be used to treat neurological symptoms caused by virus infections. This strategy will be most relevant for allo-HCT patients with drug-refractory CMV infection that lack virus-specific T cells. A recent trial using stem cell-donor- or third-party-donor-derived CMV-specific T cells for the treatment of persistent CMV infections after allo-HSCT reported complete and partial virological response rates in 62.5% and 25%, respectively (68).

Vascular complications including subarachnoid, subdural, intraparenchymal and intraventricular hemorrhages were identified by autopsy studies in the CNS of allo-HCT patients (15, 69). Low platelet counts, an altered coagulation and pre-existing vascular events are risk factors contributing to hemorrhage and thrombosis post allo-HCT (70). Microvascular injury and endothelial damage leading to increased microvascular permeability were caused by calcineurin inhibitors in patients undergoing allo-HCT (71).

Medications given pre- and post-transplant also contribute to neurological deficits in patients undergoing allo-HCT. In order to suppress the immune system of the patient and to eliminate cancer cells, patients receive conditioning therapy. The type of conditioning regimen mainly depends on the underlying disease, comorbidities and the age of the patient. Conditioning regimens can include combinations of high dose TBI with cyclophosmide and cytarabine. Reduced intensity conditioning regimen (RIC) often consist of fluradabine and busulphan and minimum dose conditioning regimens use low dose TBI and busulphan (13, 72). Cyclophosphamide induces neurotoxicity by generating reactive oxygen species which further impairs the motor coordination, learning and memory in rats (73). Busulphan, an alkylating agent, is widely used for conditioning prior to allo-HCT. Busulphan penetrates the CNS as shown by active CSF drug levels and severe CNS toxicity was observed in patients treated with this agent (74). Around 2% of the allo-HCT patients treated with busulphan were reported to develop tonic clonic seizures (75, 76). A case study reported disturbances in electroenchephalography (EEG) which lasted for about 20 days upon busulphan and cyclophosphamide treatment (77). Phenytoin is effective at preventing busulphan induced seizures (78). Chemotherapy induced toxic leukoencephalopathy has an unfavorable prognosis (79). Autopsies of patients with leukoencephalopathy revealed activation of astrocytes, infiltration of activated macrophages and a decrease in microglia expressing TMEM119 along with gliosis, demyelination in white matter (80).

In addition to neurotoxicity caused by the conditioning regimen, the GVHD prophylaxis or treatment, anti-viral drugs, antibiotics and anti-fungal agents can cause toxicity to the CNS. The calcineurin inhibitors cyclosporine A (CSA) and tacrolimus are widely used for GVHD prophylaxis as they block T cell activity (81). However the expression of calcineurin is not limited to lymphocytes, but it is also expressed by CNS cells, particularly in the hippocampus (82). In the CNS calcineurin controls the function of neurons and its blockade affects the CNS function (83). Visual disturbances, increase in the occipital lobe density, cortical abnormalities, seizures, posterior reversible encephalopathy syndrome (PRES), hallucinations, motor weakness are some of the most commonly reported side effects of CSA experienced by 10-28% of the treated patients (84–87). In line with the reports, CSA treated mixed glial cultures induced cell death of neurons and oligodendrocytes indicating drug toxicity (88). While most of the side effects induced by CSA are reversible, some reports indicate that cyclosporine induced neurotoxicity might result in long-term toxicity with permanent cortical blindness (89).The mechanism of action of tacrolimus is quite similar to CSA, while some reports suggest that CSA caused milder symptoms of neurotoxicity (50). Tacrolimus induced PRES was reported in children undergoing allo-HCT for hemoglobinopathies (90–92). Recently the JAK-1 and JAK-2 inhibitor ruxolitinib has shown activity for the treatment of corticosteroid-refractory acute and chronic GVHD (23–25). A major side effects is thrombocytopenia, which may increase the risk of cerebral hemorrhage after allo-HCT.

Antimicrobials or anti infectious drugs employed in the treatment of opportunistic infections during GVHD also pose significant threat to the CNS. Neutropenia together with encephalitis induced stroke, and vertigo are the major side effects of medications including acyclovir, gancyclovir (49). In addition, thrombocytopenia induces vascular complications ranging from subdural hematoma, hemorrhages and infarct along with increased infection rate in patients post allo-HCT (49). Amphotericin B triggers confusion, Parkinsonism, visual changes and encephalopathies in some cases (49, 93). Cefepime induced seizures, encephalopathy and myoclonus were noted in some studies (49).

In aggregate, a plethora of infections, vascular events, and drug-induced toxicities can cause neurogical symptoms that need to be ruled out before diagnosing CNS-GVHD.

## Diagnostic Procedures That Should Be Performed in Case of CNS Symptoms

The NIH Consensus Conference on criteria for clinical trials in GVHD recommends the following measures in patients with suspected CNS-GVHD (41): CSF cell count, serology, culture and polymerase chain reaction for viral, bacterial or fungal DNA. Imaging should include MRI of the CNS. MRI and CSF analysis will reveal the underlying disease of the neurological symptoms in the majority of cases. CNS-GVHD is an exclusion diagnosis meaning that other causes should be excluded before immunosuppressive therapy is started. The presence of other GVHD manifestations make the diagnosis of CNS-GVHD more likely. To exclude more rare causes for neurological symptoms such as post-transplant acute limbic encephalitis in patients with anterograde amnesia, inappropriate antidiuretic hormone secretion and EEG abnormalities, it is recommended to determine HHV-6 reactivation in the CSF and perform MRI of the brain (41). In case that clinical presentation and MRI suggest an infection, but serology and PCR from CSF remain negative a biopsy of the lesion is recommended (41). In particular when chronic fungal and viral infections as well as progressive multifocal leukoencephalopathy are suspected (94). Also if relapse of the hematological malignancy in the CNS is clinically suspected a biopsy can be considered if the CSF analysis was not conclusive.

## Summary

Despite recent advances in the clinical management of acute GVHD, CNS-GVHD is still a life threatening complication that is often difficult to diagnose. Preclinical studies have shown that allogeneic T cells infiltrate the CNS during GVHD and activate different cell types including microglia and other myeloid cells. CNS-GVHD causes damage to neurons and endothelial cells. While CNS-GVHD accounts for some of the neurological symptoms observed after allo-HCT it is important to also consider infections, vascular events, and drug-induced toxicity. Treatment of these complications e.g. reducing CSA when CSA induced neurotoxicity is suspected could exacerbate CNS-GVHD. In case of drug toxicities the responsible drugs should be changed and avoided if CNS symptoms are severe. Therefore, to improve patient outcome it is desirable to identify biomarkers that help early identification and diagnosis of CNS-GVHD in particular when other organs are not affected by GVHD.

## Author Contributions

JV and RZ developed the overall concept of this article and wrote the manuscript. All authors contributed to the article and approved the submitted version.

## Funding

The article processing charge was funded by the Baden-Wuerttemberg Ministry of Science, Research and Art and the University of Freiburg in the funding programme Open Access Publishing. This article was supported by the Deutsche Forschungsgemeinschaft (DFG, German Research Foundation) – SFB-1479 – Project ID: 441891347, SFB TRR167, SFB850 (to RZ), by the Germany’s Excellence Strategy (CIBSS – EXC-2189 – Project ID 390939984 to RZ) and by ERC Consolidator grant (681012 GvHDCure to RZ).

## Conflict of Interest

RZ received honoraria from Novartis, Incyte and Mallinckrodt.

The remaining authors declare that the research was conducted in the absence of any commercial or financial relationships that could be construed as a potential conflict of interest.​

## Publisher’s Note

All claims expressed in this article are solely those of the authors and do not necessarily represent those of their affiliated organizations, or those of the publisher, the editors and the reviewers. Any product that may be evaluated in this article, or claim that may be made by its manufacturer, is not guaranteed or endorsed by the publisher.

## References

[1] Zeiser R Blazar BR. Acute Graft-Versus-Host Disease - Biologic Process, Prevention, and Therapy. N Eng J Med (2017) 377:2167–79. doi: 10.1056/NEJMra1609337 PMC6034180 29171820

[2] Hülsdünker J Thomas OS Haring E Unger S Gonzalo Núñez N Tugues S. Immunization Against Poly-N-Acetylglucosamine Reduces Neutrophil Activation and GVHD While Sparing Microbial Diversity. Proc Natl Acad Sci USA (2019) 116:20700–6. doi: 10.1073/pnas.1908549116 PMC6789638 31527267

[3] Koyama M Mukhopadhyay P Schuster IS Henden AS Hülsdünker J Varelias A. MHC Class II Antigen Presentation by the Intestinal Epithelium Initiates Graft-Versus-Host Disease and Is Influenced by the Microbiota. Immunity (2019) 51:885–98. doi: 10.1016/j.immuni.2019.08.011 PMC6959419 31542340

[4] Hülsdünker J Ottmüller KJ Neeff H Koyama M Gao Z Thomas OS. Neutrophils Provide Cellular Communication Between Ileum and Mesenteric Lymph Nodes at Graft-Versus-Host Disease Onset. Blood (2018) 131:1858–69. doi: 10.1182/blood-2017-10-812891 PMC5909763 29463561

[5] Schwab L Goroncy L Palaniyandi S Gautam S Triantafyllopoulou A Mocsai A. Neutrophil Granulocytes Recruited Upon Translocation of Intestinal Bacteria Enhance GvHD via Tissue Damage. Nat Med (2014) 20:648–54. doi: 10.1038/nm.3517 24836575

[6] Klämbt V Wohlfeil SA Schwab L Hülsdünker J Ayata K Apostolova P. A Novel Function for P2Y2 in Myeloid Recipient-Derived Cells During GvHD. J Immunol (2015) 195:5795–804. doi: 10.4049/jimmunol.1501357 26538394

[7] Wilhelm K Ganesan J Müller T Dürr C Grimm M Beilhack A. Graft-Versus-Host Disease Enhanced by Extracellular Adenosine Triphosphate Activating P2X7R. Nat Med (2010) 12:1434–8. doi: 10.1038/nm.2242 21102458

[8] Zeiser R Penack O Holler E Idzko M. Danger Signals Activating Innate Immunity in Graft-Versus-Host Disease. J Mol Med (2011) 89:833–45. doi: 10.1007/s00109-011-0767-x 21573893

[9] Jankovic D Ganesan J Bscheider M Stickel N Weber F Guarda G. The Nlrp3-Inflammasome Regulates Acute Graft-Versus-Host Disease. J Exp Med (2013) 210:1899–910. doi: 10.1084/jem.20130084 PMC3782050 23980097

[10] Sheikh MA Toledano M Ahmed S Gul Z Hashmi SK. Noninfectious Neurologic Complications of Hematopoietic Cell Transplantation: A Systematic Review. Hematol/Oncol Stem Cell Ther (2021) 14(2):87–94. doi: 10.1016/j.hemonc.2020.05.006 32516577

[11] Das J Gill A Lo C Chan-Lam N Price S Wharton SB. A Case of Multiple Sclerosis—Like Relapsing Remitting Encephalomyelitis Following Allogeneic Hematopoietic Stem Cell Transplantation and a Review of the Published Literature. Front Immunol (2020) 11:668. doi: 10.3389/fimmu.2020.00668 32431694 PMC7214636

[12] Ke P Bao X Zhou J Zhu Q Zhuang J Hu X. Central Nervous System Complications After Allogeneic Hematopoietic Stem Cell Transplantation in Children. Acta Haematol (2019) 142(4):217–23. doi: 10.1159/000499651 31597154

[13] Siegal D Keller A Xu W Bhuta S Kim DH Kuruvilla J. Central Nervous System Complications After Allogeneic Hematopoietic Stem Cell Transplantation: Incidence, Manifestations, and Clinical Significance. Biol Blood Marrow Transplant (2007) 13(11):1369–79. doi: 10.1016/j.bbmt.2007.07.013 17950923

[14] Barba P Piñana JL Valcárcel D Querol L Martino R Sureda A. Early and Late Neurological Complications After Reduced-Intensity Conditioning Allogeneic Stem Cell Transplantation. Biol Blood Marrow Transplant (2009) 15(11):1439–46. doi: 10.1016/j.bbmt.2009.07.013 19822304

[15] Bleggi-Torres L De Medeiros B Werner B Neto J Loddo G Pasquini R. Neuropathological Findings After Bone Marrow Transplantation: An Autopsy Study of 180 Cases. Bone Marrow Transplant (2000) 25(3):301–7. doi: 10.1038/sj.bmt.1702140 10673702

[16] Hartrampf S Dudakov JA Johnson LK Smith OM Tsai J Singer NV. The Central Nervous System is a Target of Acute Graft Versus Host Disease in Mice. Blood (2013) 121:1906–10. doi: 10.1182/blood-2012-09-456590 PMC3591808 23299314

[17] Kaliyaperumal S Watkins B Sharma P Furlan S Ramakrishnan S Giver C. CD8-Predominant T-Cell CNS Infiltration Accompanies GVHD in Primates and Is Improved With Immunoprophylaxis. Blood (2014) 123:1967–9. doi: 10.1182/blood-2014-01-547612 PMC3962171 24652969

[18] Hickey WF Kimura H. Graft-Vs.-Host Disease Elicits Expression of Class I and Class II Histocompatibility Antigens and the Presence of Scattered T Lymphocytes in Rat Central Nervous System. Proc Natl Acad Sci (1987) 84(7):2082–6. doi: 10.1073/pnas.84.7.2082 PMC304589 3550805

[19] Sedgwick JD Ford AL Foulcher E Airriess R. Central Nervous System Microglial Cell Activation and Proliferation Follows Direct Interaction With Tissue-Infiltrating T Cell Blasts. J Immunol (1998) 160(11):5320–30. 9605131

[20] Bachiller S Jiménez-Ferrer I Paulus A Yang Y Swanberg M Deierborg T. Microglia in Neurological Diseases: A Road Map to Brain-Disease Dependent-Inflammatory Response. Front Cell Neurosci (2018) 12:488. doi: 10.3389/fncel.2018.00488 30618635 PMC6305407

[21] Belle L Koester E Hansen E Lawlor M Hillard C Drobyski WR. Host Interleukin 6 and Indoleamine 2,3 Dioxygenase Regulate Inflammation in the Brain During Graft Versus Host Disease. Blood (2016) 128(22):2145–5. doi: 10.1182/blood.v128.22.2145.2145

[22] Belle L Zhou V Stuhr KL Beatka M Siebers EM Knight JM. Host Interleukin 6 Production Regulates Inflammation But Not Tryptophan Metabolism in the Brain During Murine GVHD. JCI Insight (2017) 2(14):e93726. doi: 10.1172/jci.insight.93726 PMC5518565 28724796

[23] Zeiser R Polverelli N Ram R Hashmi SK Chakraverty R Middeke JM. Ruxolitinib for Glucocorticoid-Refractory Chronic Graft-Versus-Host Disease. N Eng J Med (2021) 385:228–38. doi: 10.1056/NEJMoa2033122 34260836

[24] Zeiser R von Bubnoff N Butler J Mohty M Niederwieser D Or R. Ruxolitinib for Glucocorticoid-Refractory Acute Graft-Versus-Host Disease. N Eng J Med (2020) 382:1800–10. doi: 10.1056/NEJMoa1917635 32320566

[25] Zeiser R Burchert A Lengerke C Verbeek M Maas-Bauer K Metzelder SK. Ruxolitinib in Corticosteroid-Refractory Graft-Versus-Host Disease After Allogeneic Stem Cell Transplantation: A Multi-Center Survey. Leukemia (2015) 29:2062–8. doi: 10.1038/leu.2015.212 PMC4854652 26228813

[26] Mathew NR Vinnakota JM Apostolova P Erny D Hamarsheh S Andrieux G. Graft-Versus-Host Disease of the CNS Is Mediated by TNF Upregulation in Microglia. J Clin Invest (2020) 130:1315–29. doi: 10.1172/JCI130272 PMC7269577 31846439

[27] Furukawa H Yamashita A del Rey A Besedovsky H. C-Fos Expression in the Rat Cerebral Cortex During Systemic GvH Reaction. Neuroimmunomodulation (2004) 11(6):425–33. doi: 10.1159/000080154 15467359

[28] Unger ER Sung JH Manivel JC Chenggis ML Blazar BR Krivit W. Male Donor-Derived Cells in the Brains of Female Sexmismatched Bone Marrow Transplant Recipients: A Y-Chromosome Specific in Situ Hybridization Study. J Neuropathol Exp Neurol (1993) 52:460–70. doi: 10.1097/00005072-199309000-00004 8103085

[29] Mariotti J Penack O Castagna L. Acute Graft-Versus-Host-Disease Other Than Typical Targets: Between Myths and Facts. Transplant Cell Ther (2021) 27(2):115–24. doi: 10.1016/j.bbmt.2020.09.033 33017661

[30] Ruggiu M Cuccuini W Mokhtari K Meignin V Peffault de Latour R Robin M. Case Report: Central Nervous System Involvement of Human Graft Versus Host Disease: Report of 7 Cases and a Review of Literature. Medicine (2017) 96(42):e8303–3. doi: 10.1097/MD.0000000000008303 PMC5662398 29049232

[31] Shortt J Hutton E Faragher M Spencer A. Central Nervous System Graft-Versus-Host Disease Post Allogeneic Stem Cell Transplant. Br J Haematol (2006) 132:245–7. doi: 10.1111/j.1365-2141.2005.05864.x 16398660

[32] Saad AG Alyea EP Wen PY DeGirolami U Kesari S. Graft-Versus-Host Disease of the CNS After Allogeneic Bone Marrow Transplantation. J Clin Oncol (2009) 27:147–9. doi: 10.1200/JCO.2009.21.7919 19667266

[33] Li M Zhang Y Guan Y Zhang Z Dong H Zhao Y. A Case Report of Central Nervous System Graft-Versus-Host Disease and Literature Review. Front Neurol (2021) 12:621392(327). doi: 10.3389/fneur.2021.621392 33776885 PMC7987907

[34] Blasiak KP Simonetta F Vargas M-I Chalandon Y. Central Nervous System Graft-Versus-Host Disease (CNS-GvHD) After Allogeneic Haematopoietic Stem Cell Transplantation. Case Rep (2018) 2018:bcr–2017-221840. doi: 10.1136/bcr-2017-221840 PMC5780588 29330269

[35] Bonnan M Gianoli-Guillerme M Courtade H Demasles S Krim E Marasescu R. Estimation of Intrathecal IgG Synthesis: Simulation of the Risk of Underestimation. Ann Clin Transl Neurol (2018) 5(5):524–37. doi: 10.1002/acn3.548 PMC5945966 29761116

[36] Lyu H-R He X-Y Hao H-J Lu W-Y Jin X Zhao Y-J. Noninvasive Tools Based on Immune Biomarkers for the Diagnosis of Central Nervous System Graft-vs-Host Disease: Two Case Reports and a Review of the Literature. World J Clin cases (2021) 9(6):1359–66. doi: 10.12998/wjcc.v9.i6.1359 PMC7896680 33644203

[37] Zhang XH Zhao X Wang CC Han W Chen H Chen YH. IgG Synthesis Rate and Anti-Myelin Oligodendrocyte Glycoprotein Antibody in CSF may be Associated With the Onset of CNS Demyelination After Haplo-HSCT. Ann Hematol (2018) 97(8):1399–406. doi: 10.1007/s00277-018-3299-4 29568992

[38] Ruggiu M Cuccuini W Mokhtari K Meignin V Latour R Robin M. Case Report: Central Nervous System Involvement of Human Graft Versus Host Disease. Medicine (2017) 96:e8303. doi: 10.1097/MD.0000000000008303 29049232 PMC5662398

[39] Hümmert MW Stadler M Hambach L Gingele S Bredt M Wattjes MP. Severe Allo-Immune Antibody-Associated Peripheral and Central Nervous System Diseases After Allogeneic Hematopoietic Stem Cell Transplantation. Sci Rep (2021) 11:8527. doi: 10.1038/s41598-021-87989-z 33875720 PMC8055885

[40] Das J Gill A Lo C Chan-Lam N Price S Wharton SB. A Case of Multiple Sclerosis-Like Relapsing Remitting Encephalomyelitis Following Allogeneic Hematopoietic Stem Cell Transplantation and a Review of the Published Literature. Front Immunol (2020) 11:668. doi: 10.3389/fimmu.2020.00668 32431694 PMC7214636

[41] Grauer O Wolff D Bertz H Greinix H Kühl JS Lawitschka A. Neurological Manifestations of Chronic Graft-Versus-Host Disease After Allogeneic Haematopoietic Stem Cell Transplantation: Report From the Consensus Conference on Clinical Practice in Chronic Graft-Versus-Host Disease. Brain (2010) 133:2852–65. doi: 10.1093/brain/awq245 20846944

[42] Miller HK Braun TM Stillwell T Harris AC Choi S Connelly J. Infectious Risk After Allogeneic Hematopoietic Cell Transplantation Complicated by Acute Graft-Versus-Host Disease. Biol Blood Marrow Transplant J Am Soc Blood Marrow Transplant (2017) 23(3):522–8. doi: 10.1016/j.bbmt.2016.12.630 PMC5551893 28017733

[43] Sakellari I Gavriilaki E Papagiannopoulos S Gavriilaki M Batsis I Mallouri D. Neurological Adverse Events Post Allogeneic Hematopoietic Cell Transplantation: Major Determinants of Morbidity and Mortality. J Neurol (2019) 266(8):1960–72. doi: 10.1007/s00415-019-09372-3 31087160

[44] Dietrich U Hettmann M Maschke M Doerfler A Schwechheimer K Forsting M. Cerebral Aspergillosis: Comparison of Radiological and Neuropathologic Findings in Patients With Bone Marrow Transplantation. Eur Radiol (2001) 11(7):1242–9. doi: 10.1007/s003300000756 11471618

[45] Anciones C de Felipe A de Albóniga-Chindurza A Acebrón F Pián H Masjuán J. Acute Stroke as First Manifestation of Cerebral Aspergillosis. J Stroke Cerebrovascular Dis (2018) 27(11):3289–93. doi: 10.1016/j.jstrokecerebrovasdis.2018.07.031 30172679

[46] Lai PH Lin SM Pan HB Yang CF. Disseminated Miliary Cerebral Candidiasis. AJNR Am J Neuroradiol (1997) 18(7):1303–6. PMC8338024 9282859

[47] Martino R Maertens J Bretagne S Rovira M Deconinck E Ullmann A. Toxoplasmosis After Hematopoietic Stem Cell Transplantation. Clin Infect Dis (2000) 31(5):1188–94. doi: 10.1086/317471 11073751

[48] Fricker-Hidalgo H Bulabois C-E Brenier-Pinchart M-P Hamidfar R Garban F Brion J-P. Diagnosis of Toxoplasmosis After Allogeneic Stem Cell Transplantation: Results of DNA Detection and Serological Techniques. Clin Infect Dis (2009) 48(2):e9–15. doi: 10.1086/595709 19072243

[49] Dulamea AO Lupescu IG. Neurological Complications of Hematopoietic Cell Transplantation in Children and Adults. Neural regener Res (2018) 13(6):945–54. doi: 10.4103/1673-5374.233431 PMC6022482 29926815

[50] Ayas M Al-Jefri A Al-Seraihi A. In Cyclosporine Induced Neurotoxicity, Is Tacrolimus an Appropriate Substitute or Is It Out of the Frying Pan and Into the Fire? Pediatr Blood Cancer (2008) 50(2):426; author reply 427–6. doi: 10.1002/pbc.21211 17427233

[51] Avivi I Wittmann T Henig I Kra-Oz Z Szwarcwort Cohen M Zuckerman T. Development of Multifocal Leukoencephalopathy in Patients Undergoing Allogeneic Stem Cell Transplantation-Can Preemptive Detection of John Cunningham Virus Be Useful? Int J Infect Dis (2014) 26:107–9. doi: 10.1016/j.ijid.2014.03.1381 25038519

[52] Wüthrich C Kesari S Kim WK Williams K Gelman R Elmeric D. Characterization of Lymphocytic Infiltrates in Progressive Multifocal Leukoencephalopathy: Co-Localization of CD8(+) T Cells With JCV-Infected Glial Cells. J Neurovirol (2006) 12(2):116–28. doi: 10.1080/13550280600716604 16798673

[53] Ogata M Fukuda T Teshima T. Human Herpesvirus-6 Encephalitis After Allogeneic Hematopoietic Cell Transplantation: What We Do and Do Not Know. Bone Marrow Transplant (2015) 50(8):1030–6. doi: 10.1038/bmt.2015.76 25915811

[54] Zerr DM. Human Herpesvirus 6 and Central Nervous System Disease in Hematopoietic Cell Transplantation. J Clin Virol (2006) 37(Suppl 1):S52–6. doi: 10.1016/s1386-6532(06)70012-9 17276370

[55] Zerr DM Fann JR Breiger D Boeckh M Adler AL Xie H. HHV-6 Reactivation and Its Effect on Delirium and Cognitive Functioning in Hematopoietic Cell Transplantation Recipients. Blood J Am Soc Hematol (2011) 117(19):5243–9. doi: 10.1182/blood-2010-10-316083 PMC3109545 21389320

[56] Inui Y Yakushijin K Okamura A Tanaka Y Shinzato I Nomura T. Human Herpesvirus 6 Encephalitis in Patients Administered Mycophenolate Mofetil as Prophylaxis for Graft-Versus-Host Disease After Allogeneic Hematopoietic Stem Cell Transplantation. Transplant Infect Dis (2019) 21(1):e13024. doi: 10.1111/tid.13024 30414316

[57] Zivković S. Neuroimaging and Neurologic Complications After Organ Transplantation. J Neuroimaging (2007) 17(2):110–23. doi: 10.1111/j.1552-6569.2007.00097.x 17441832

[58] Dharnidharka VR Webster AC Martinez OM Preiksaitis JK Leblond V Choquet S. Post-Transplant Lymphoproliferative Disorders. Nat Rev Dis Primers (2016) 2:15088. doi: 10.1038/nrdp.2015.88 27189056

[59] Uhlin M Wikell H Sundin M Blennow O Maeurer M Ringden O. Risk Factors for Epstein-Barr Virus-Related Post-Transplant Lymphoproliferative Disease After Allogeneic Hematopoietic Stem Cell Transplantation. Haematologica (2014) 99(2):346–52. doi: 10.3324/haematol.2013.087338 PMC3912966 24056821

[60] Pickhardt PJ Wippold FJ. 2nd: Neuroimaging in Posttransplantation Lymphoproliferative Disorder. AJR Am J Roentgenol (1999) 172(4):1117–21. doi: 10.2214/ajr.172.4.10587158 10587158

[61] Zeiser R Grüllich C Bertz H Pantazis G Hufert FT Bley TA. Late Cytomegalovirus Polyradiculopathy Following Haploidentical CD34+-Selected Hematopoietic Stem Cell Transplantation. Bone Marrow Transplant (2004) 33:243–5. doi: 10.1038/sj.bmt.1704311 14716290

[62] Reddy SM Winston DJ Territo MC Schiller GJ. CMV Central Nervous System Disease in Stem-Cell Transplant Recipients: An Increasing Complication of Drug-Resistant CMV Infection and Protracted Immunodeficiency. Bone Marrow Transplant (2010) 45(6):979–84. doi: 10.1038/bmt.2010.35 20190836

[63] Sarva H Graber J Remanan R Rosenblum M Omuro A. CMV Encephalitis in BMT Recipients. Bone Marrow Transplant (2012) 47(2):318–20. doi: 10.1038/bmt.2011.80 21460874

[64] Ke P Bao X Zhou J Li X Zhuang J He X. Donor CMV-Specific Cytotoxic T Lymphocytes Successfully Treated Drug-Resistant Cytomegalovirus Encephalitis After Allogeneic Hematopoietic Stem Cell Transplantation. Hematology (2020) 25(1):43–7. doi: 10.1080/16078454.2019.1710945 31906810

[65] Kaeuferle T Krauss R Blaeschke F Willier S Feuchtinger T. Strategies of Adoptive T -Cell Transfer to Treat Refractory Viral Infections Post Allogeneic Stem Cell Transplantation. J Hematol Oncol (2019) 12:13. doi: 10.1186/s13045-019-0701-1 30728058 PMC6364410

[66] Bollard CM Heslop HE. T Cells for Viral Infections After Allogeneic Hematopoietic Stem Cell Transplant. Blood (2016) 127:3331–40. doi: 10.1182/blood-2016-01-628982 PMC4929925 27207801

[67] Einsele H Ljungman P Boeckh M. How I Treat CMV Reactivation After Allogeneic Hematopoietic Stem Cell Transplantation. Blood (2020) 135:1619–29. doi: 10.1182/blood.2019000956 PMC7484743 32202631

[68] Neuenhahn M Albrecht J Odendahl M Schlott F Dössinger G Schiemann M. Transfer of Minimally Manipulated CMV-Specific T Cells From Stem Cell or Third-Party Donors to Treat CMV Infection After Allo-HSCT. Leukemia (2017) 10:2161–71. doi: 10.1038/leu.2017.16 28090089

[69] Taylor JW Schiff D. Neurologic Complications. Germany: Springer International Publishing (2015). doi: 10.1007/978-3-319-13832-9_25

[70] Balaguer-Rosello A Bataller L Piñana JL Montoro J Lorenzo I Villalba A. Noninfectious Neurologic Complications After Allogeneic Hematopoietic Stem Cell Transplantation. Biol Blood Marrow Transplant (2019) 25:1818–24. doi: 10.1016/j.bbmt.2019.05.024 31132454

[71] Nishiguchi T Mochizuki K Shakudo M Takeshita T Hino M Inoue Y. CNS Complications of Hematopoietic Stem Cell Transplantation. AJR Am J Roentgenol (2009) 192(4):1003–11. doi: 10.2214/ajr.08.1787 19304707

[72] Quant E Wen PY. Neurological Complications of Hematopoietic Stem Cell Transplantation. In: Cancer Neurology In Clinical Practice. Germany: Springer (2008).

[73] Singh S Kumar A. Protective Effect of Edaravone on Cyclophosphamide Induced Oxidative Stress and Neurotoxicity in Rats. Curr Drug Saf (2019) 14(3):209–16. doi: 10.2174/1574886314666190506100717 PMC6864589 31057112

[74] Vassal G Deroussent A Hartmann O Challine D Benhamou E Valteau-Couanet D. Dose-Dependent Neurotoxicity of High-Dose Busulfan in Children: A Clinical and Pharmacological Study. Cancer Res (1990) 50(19):6203–7. 2400986

[75] McCune JS Holmberg LA. Busulfan in Hematopoietic Stem Cell Transplant Setting. Expert Opin Drug Metab Toxicol (2009) 5(8):957–69. doi: 10.1517/17425250903107764 19611402

[76] Caselli D Rosati A Faraci M Podda M Ripaldi M Longoni D. Risk of Seizures in Children Receiving Busulphan-Containing Regimens for Stem Cell Transplantation. Biol Blood Marrow Transplant (2014) 20(2):282–5. doi: 10.1016/j.bbmt.2013.10.028 24201160

[77] La Morgia C Mondini S Guarino M Bonifazi F Cirignotta F. Busulfan Neurotoxicity and EEG Abnormalities: A Case Report. Neurol Sci (2004) 25(2):95–7. doi: 10.1007/s10072-004-0237-0 15221628

[78] Eberly AL Anderson GD Bubalo JS McCune JS. Optimal Prevention of Seizures Induced by High-Dose Busulfan. Pharmacother: J Hum Pharmacol Drug Ther (2008) 28(12):1502–10. doi: 10.1592/phco.28.12.1502 19025431

[79] Moore-Maxwell CA Datto MB Hulette CM. Chemotherapy-Induced Toxic Leukoencephalopathy Causes a Wide Range of Symptoms: A Series of Four Autopsies. Modern Pathol (2004) 17(2):241–7. doi: 10.1038/modpathol.3800049 14704718

[80] Park S-H Lim KY Kim S-I Kim H Kang J Park JW. Toxic Leukoencephalopathy Caused by Chemotherapeutic Drugs Other Than Methotrexate. Research Square Platform LLC (2021). doi: 10.21203/rs.3.rs-36667/v1 PMC9347126 35922754

[81] Zeiser R Nguyen VH Beilhack A Buess M Schulz S Baker J. Inhibition of CD4+CD25+ Regulatory T Cell Function by Calcineurin Dependent Interleukin-2 Production. Blood (2006) 108:390–9. doi: 10.1182/blood-2006-01-0329 PMC1895845 16522809

[82] Klee C Draetta G Hubbard M. Calcineurin. Adv Enzymol (1988) 61:149–200. doi: 10.1002/9780470123072.ch4 2833077

[83] Tan TC Robinson PJ. Mechanisms of Calcineurin Inhibitor-Induced Neurotoxicity. Transplant Rev (2006) 20(1):49–60. doi: 10.1016/j.trre.2006.02.005

[84] Straathof K Anoop P Allwood Z Silva J Nikolajeva O Chiesa R. Long-Term Outcome Following Cyclosporine-Related Neurotoxicity in Paediatric Allogeneic Haematopoietic Stem Cell Transplantation. Bone Marrow Transplant (2017) 52(1):159–62. doi: 10.1038/bmt.2016.232 PMC5220133 27643866

[85] Takahata M Hashino S Izumiyama K Chiba K Suzuki S Asaka M. Cyclosporin A-Induced Encephalopathy After Allogeneic Bone Marrow Transplantation With Prevention of Graft-Versus-Host Disease by Tacrolimus. Bone Marrow Transplant (2001) 28(7):713–5. doi: 10.1038/sj.bmt.1703221 11704797

[86] Bechstein WO. Neurotoxicity of Calcineurin Inhibitors: Impact and Clinical Management. Transplant Int (2000) 13(5):313–26. doi: 10.1111/j.1432-2277.2000.tb01004.x 11052266

[87] Maffini E Festuccia M Brunello L Boccadoro M Giaccone L Bruno B. Neurologic Complications After Allogeneic Hematopoietic Stem Cell Transplantation. Biol Blood Marrow Transplant (2017) 23(3):388–97. doi: 10.1016/j.bbmt.2016.12.632 28039081

[88] McDonald JW Goldberg MP Gwag BJ Chi SI Choi DW. Cyclosporine Induces Neuronal Apoptosis and Selective Oligodendrocyte Death in Cortical Cultures. Ann Neurol: Off J Am Neurol Assoc Child Neurol Soc (1996) 40(5):750–8. doi: 10.1002/ana.410400511 8957016

[89] Casanova B Prieto M Deya E Gisbert C Mir J Berenguer J. Persistent Cortical Blindness After Cyclosporine Leukoencephalopathy. Liver Transplant Surg (1997) 3(6):638–40. doi: 10.1002/lt.500030614 9404966

[90] Noè A Cappelli B Biffi A Chiesa R Frugnoli I Biral E. High Incidence of Severe Cyclosporine Neurotoxicity in Children Affected by Haemoglobinopaties Undergoing Myeloablative Haematopoietic Stem Cell Transplantation: Early Diagnosis and Prompt Intervention Ameliorates Neurological Outcome. Ital J Pediatr (2010) 36(1):1–6. doi: 10.1186/1824-7288-36-1 20181110 PMC2829572

[91] Thompson D Harrington Y de la Fuente J. The Incidence of Posterior Reversible Encephalopathy Syndrome Is Increased in BMT for Haemoglobinopathies. Blood (2013) 122(21):4579. doi: 10.1182/blood.V122.21.4579.4579

[92] Gaziev J Marziali S Paciaroni K Isgrò A Di Giuliano F Rossi G. Posterior Reversible Encephalopathy Syndrome After Hematopoietic Cell Transplantation in Children With Hemoglobinopathies. Biol Blood Marrow Transplant (2017) 23(9):1531–40. doi: 10.1016/j.bbmt.2017.05.033 28602890

[93] Duléry R. Neurological Complications. Germany: Springer International Publishing (2019), 403–407. doi: 10.1007/978-3-030-02278-5_53 32091806

[94] Kharfan-Dabaja MA Ayala E Greene J Rojiani A Murtagh FR Anasetti C. Two Cases of Progressive Multifocal Leukoencephalopathy After Allogeneic Hematopoietic Cell Transplantation and a Review of the Literature. Bone Marrow Transplant (2007) 39:101–7. doi: 10.1038/sj.bmt.1705548 17143300

